# GlycA: Evaluation of a New Biomarker of Acute Pancreatitis

**DOI:** 10.3390/biom13101530

**Published:** 2023-10-16

**Authors:** Ishani Shah, William Yakah, Awais Ahmed, Steven D. Freedman, Zhenghui G. Jiang, Sunil G. Sheth

**Affiliations:** 1 Division of Gastroenterology and Hepatology, Department of Medicine, Beth Israel Deaconess Medical Center, Harvard Medical School, Boston, MA 02115, USA; 2 Institute of Human Nutrition, Columbia University Medical Center, New York, NY 10032, USA

**Keywords:** acute pancreatitis, biomarker, GlycA, nuclear magnetic resonance

## Abstract

Background: Acute pancreatitis (AP) is a leading cause of gastrointestinal hospital admissions, with up to 40% mortality in patients with moderate–severe AP. Glycoprotein acetylation (GlycA) is measured as a nuclear magnetic resonance signal (NMR) of the post-translational modification of glycosylated acute-phase proteins released during inflammation. We aimed to investigate the role of GlycA as an inflammatory biomarker of AP. Methods: We prospectively enrolled 20 AP patients and 22 healthy controls and collected EDTA plasma samples at admission and discharge. NMR spectra were acquired from these samples using a 400 MHz Vantera^®^ Clinical Analyzer, and GlycA concentrations were calculated (normal = 400 μmol/L). The GlycA NMR signal, at 2.00 ± 0.01 ppm in the NMR spectrum, is derived from the N-acetyl methyl group protons within the carbohydrate side chains of circulating glycoproteins such as α1-acid glycoprotein, haptoglobin, α1-antitrypsin, α1-antichymotrypsin, and transferrin. GlycA levels were then compared between AP patients and controls, as well as within the AP group, based on etiology and severity. Results: Demographic comparisons were similar, except for a higher BMI in AP patients compared to healthy controls (29.9 vs. 24.8 kg/m^2^; *p* < 0.001). AP was mild in 10 patients, moderate in 7, and severe in 3. GlycA levels were higher in AP patients than healthy controls on admission (578 vs. 376 μmol/L, *p* < 0.001) and at discharge (655 vs. 376 μmol/L, *p* < 0.001). GlycA levels were significantly higher in patients with moderate–severe AP than in those with mild AP at discharge (533 vs. 757 μmol/L, *p* = 0.023) but not at admission. After adjusting for BMI, multivariable regression indicated that patients with GlycA levels > 400 μmol/L had significantly higher odds of having AP of any severity (OR = 6.88; 95% CI, 2.07–32.2; *p* = 0.004) and mild AP (OR = 6.12; 95% CI, 1.48–42.0; *p* = 0.025) than controls. Conclusion: Our pilot study highlights the use of GlycA as a novel diagnostic biomarker of inflammation in patients with AP. Our study shows that GlycA levels were significantly higher in hospitalized AP patients compared to healthy controls. Patients with moderate-to-severe AP had higher GlycA levels compared to patients with mild AP at the time of their hospital discharge, suggesting persistent inflammation in patients with severe disease.

## 1. Introduction

Acute pancreatitis (AP) is one of the most common gastrointestinal causes of hospitalization, resulting in a significant economic burden to the US healthcare system [1,2,3,4]. While most patients experience a mild disease course, about 20% of patients have moderate-to-severe disease characterized by pancreatic necrosis and varying degrees of systemic organ failure, which is often associated with high mortality (20–40%) [2,5,6]. Over the past few decades, in efforts to assess disease activity and severity in patients with AP, several clinical and biochemical scoring systems as well as individual biomarkers have been proposed, such as the C-reactive protein (CRP) level, Bedside Index of Severity in Acute Pancreatitis (BISAP) score, Acute Physiology and Chronic Health Evaluation (APACHE-II) score, Ranson score, Glasgow score, systemic inflammatory response syndrome (SIRS) score, and Pancreatitis Activity Scoring System (PASS) score [7,8,9,10]. However, most of these lack diagnostic accuracy and are not routinely used in clinical practice [11,12,13,14].

The pathophysiology of AP is complex and primarily driven by macrophage-derived pro-inflammatory cytokines such as interleukin-1 (IL-1), IL-6, and tumor necrosis factor-alpha (TNF-α), which are known to enter the blood stream and cause a systemic inflammatory response, including the hepatic production of acute phase reactants. Most of these acute-phase reactants are N-linked glycoproteins that contain oligosaccharide chains connected to the side-chain nitrogen group of their asparagine residues. As the inflammatory response evolves, these N-glycan oligosaccharides further increase in number and composition (i.e., further extension and branching of glycans) [15]. In this context, the nuclear magnetic resonance (NMR) signal from the N-acetyl glucosamine residues within the carbohydrate side chains of circulating proteins can be measured and has been developed as a novel biomarker of inflammation, called GlycA, which can be assessed in serum or plasma. Since this NMR signal arises from circulating glycoproteins, GlycA is a composite measure of both the number and complexity of N-glycan side chains in acute-phase glycoproteins such as α1-acid glycoprotein, haptoglobin, α1-antitrypsin, and α1-antichymotrypsin [16,17]. The role of GlycA has been extensively studied in cardiovascular diseases as well as acute inflammatory conditions such as sepsis, Kawasaki’s disease, and chronic inflammatory diseases such as rheumatoid arthritis, systemic lupus erythematosus, psoriasis, and inflammatory bowel disease (IBD); however, GlycA has not been studied in AP [18,19,20,21].

Therefore, the aim of our study was to investigate GlycA in patients with AP compared to healthy controls. We also sought to study the correlation of GlycA with the etiology and severity of AP.

## 2. Methods

### 2.1. Study Design and Patient Selection

Patients with AP presenting to the Beth Israel Deaconess Medical Center emergency department (ED) between July 2020 and June 2021 were prospectively enrolled in our study. AP was diagnosed based on meeting two of the three revised Atlanta criteria: (1) abdominal pain consistent with AP; (2) serum lipase activity at least three times greater than the upper limit of normal; and (3) characteristic AP findings on cross-sectional imaging such as computed tomography (CT) or magnetic resonance imaging (MRI) [6]. The exclusion criteria included age less than 18 years or greater than 85 years, a known diagnosis of chronic pancreatitis, and a prior history of pancreatic surgery. We obtained written informed consent for participation in the study from eligible patients within the first 24 h of their presentation. Blood samples were collected from each of the enrolled patients at two different time points of their hospitalization: the first blood sample was collected within the first 24 h of presentation to the ED, and the second blood sample was collected on the day of discharge from the hospital. This single-center study was approved by the Institutional Review Board of Beth Israel Deaconess Medical Center.

### 2.2. Healthy Controls

Subjects who were apparently healthy (internal) were also enrolled as controls in our study (*n* = 22). The eligibility criteria for healthy controls were age >18 years, absence of known pancreatic disease, and absence of other chronic comorbidities such as diabetes, hypertension, heart disease, chronic liver disease, chronic kidney disease, chronic lung disease, or malignancy. Informed consent was obtained, followed by the collection of one fasting blood sample from each healthy control. The same protocols were followed for both sample collection and processing in healthy controls and enrolled AP patients. Basic demographic data (age, sex, race, and BMI) were obtained for each healthy control.

In addition, subjects who were apparently healthy (*n* = 477) were also selected from a study performed at LipoScience, Raleigh, NC, USA (now Labcorp) (external healthy controls). This population included adults 18 to 84 years of age. Those with a history of coronary artery disease, cerebrovascular accident, chronic kidney disease, heart failure, type 2 diabetes mellitus, hypertension, and obesity (BMI ≥ 30 kg/m2) were excluded. In addition, samples for the healthy cohort were chosen to be age and sex matched with the subjects in the AP cohort. NMR testing of serum samples was performed in 2015, and the stored NMR spectra were used to quantify GlycA. Additional details for this apparently healthy population have been previously reported [22]. The study protocol was approved by the Chesapeake IRB, Morrisville, NC, USA (Pro00001317, 2012). The study was conducted in accordance with the Declaration of Helsinki guidelines, and all patients provided written informed consent.

### 2.3. Data Collection

We collected the following information for all enrolled patients: demographic and clinical characteristics, laboratory and radiologic findings, and data on complications as well as procedures performed during the hospital course. Individual variables included age, sex, race, body mass index (BMI), alcohol use, smoking status, diabetes status, presence of prior AP, etiology of AP, severity of AP (mild, moderate, or severe based on the revised Atlanta criteria), presence of pancreatic necrosis and other local complications, inpatient mortality, time taken to initiate refeeding after initial nil per os (or nothing by mouth), admission to the intensive care unit (ICU), and length of stay (LOS).

### 2.4. GlycA Measurement

Fasting and non-fasting blood samples were collected in purple-top EDTA plasma specimen tubes. EDTA plasma was prepared according to the manufacturer’s directions, and samples were stored at <−70 °C until testing. NMR spectra were acquired from EDTA plasma samples using a 400 MHz Vantera^®^ Clinical Analyzer (Labcorp, Morrisville, NC, USA), and GlycA concentrations were calculated as previously described [16,17,22]. The GlycA NMR signal, at 2.00 ± 0.01 ppm in the NMR spectrum, is derived from the N-acetyl methyl group protons within the carbohydrate side chains of circulating glycoproteins such as α1-acid glycoprotein, haptoglobin, α1-antitrypsin, α1-antichymotrypsin, and transferrin [16]. The coefficients of variation (%CV) for the GlycA assay range from 1.2 to 2.3%. GlycA measurements have been shown to be stable for at least 12 years if measured from plasma stored frozen at <−70 °C.

### 2.5. Statistical Analysis

Categorical variables are presented as proportions, and continuous variables as the mean with standard deviation (SD). Hypothesis testing was performed using the Pearson 2 test for categorical variables and the Student’s *t*-test for continuous variables. Logistic regression following a multivariable linear regression model was used to determine the association of several clinical outcomes of AP (ICU stay, necrosis, severity, etc.) with GlycA levels (categorized with a cutoff of >400 μmol/L). All analyses were performed using R software (version 3.6.1, R Core Team 2018a, Vienna, Austria) within RStudio (version 1.1463, Rstudio, Inc., Vienna, Austria) using the tidyverse (Wickham, 2017) package. *p* < 0.05 was considered statistically significant.

## 3. Results

### 3.1. Description of the Study Population

A total of 20 patients with AP were enrolled in this study over a one-year period. There was no significant difference in the mean age of AP patients compared to internal and external healthy controls (41.8 ± 11.6 vs. 39.9 ± 12.8 years, *p* = 0.49) (Table 1). Compared to healthy controls, patients with AP had a higher BMI (29.9 vs. 24.8 kg/m^2^; *p* < 0.001), but there were no differences in sex, race, or other major demographic characteristics. Regarding AP etiology, 50% (10/20) of patients had alcohol-related AP, 15% (3/20) had hypertriglyceridemia-related AP, 15% (3/20) had biliary AP, and 20% (4/20) had AP of other etiologies. Among the latter four patients, two had autoimmune AP (type II), one had idiopathic AP, and one had iatrogenic AP. Of all patients, 50% presented with their first episode of AP, while 50% had a history of prior AP.

Regarding AP severity, 50% (10/20) of patients had mild AP, 35% (7/20) had moderate AP, and 15% (3/20) had severe AP. The 10 patients with mild AP did not have any evidence of local pancreatic complications or end-organ damage. Of the seven patients with moderate AP, two met the criteria for moderate AP based on temporary end-organ damage only, while the other five patients with moderate AP had both temporary end-organ damage (acute kidney injury) and local pancreatic complications in the form of pancreatic fluid collections (with and without necrosis). The diagnosis of severe AP in the remaining three patients was based on the presence of necrotic pancreatic collections, prolonged SIRS criteria, and persistent end-organ damage beyond 48 h (acute kidney injury requiring renal replacement therapy, vasopressor-dependent shock, and hypoxic respiratory failure requiring external oxygen support). 

Two or more of the SIRS criteria were met in all patients with moderate AP (for less than 48 h), while SIRS persisting beyond 48 h was observed in all three patients with severe AP. None of the patients with mild AP met the SIRS criteria.

All 20 patients with AP received treatment as per the standard of care, which includes intravenous fluid resuscitation, pain control, and nutritional optimization. Of the 10 patients with moderate-to-severe AP, only one patient with severe AP required invasive treatment in the form of pancreatic fluid collection drainage due to superimposed infection; the remainder were treated supportively. 

In terms of treatment outcomes, the mean time to initiation of refeeding in the entire cohort was 1.45 ± 1.0 days, while the mean LOS was 5.7 ± 5.1 days. None of the enrolled patients died during hospitalization. 

### 3.2. Sample Availability

Notably, we collected 20 samples from each of the enrolled patients at the time of admission. Of the samples collected and sent for NMR testing, the GlycA levels of three samples could not be processed, as the specimen integrity was found to be suboptimal at the time of sample processing. This was attributed to potential suboptimal temperature conditions during storage and transfer. These three admission samples belonged to patients with moderate AP (two patients) and severe AP (one patient). We also collected discharge specimens from 16 out of the 20 enrolled patients. Of the four patients whose discharge samples were not collected, one patient had left the hospital against medical advice, another patient refused blood draw on the day of discharge, and the other two patients were discharged within 24 h of admission (i.e., within 48 h of ED presentation), and therefore, a second sample was not collected (since <24 h had elapsed between admission sample collection and discharge). Three of these patients belonged to the mild AP cohort, and the patient who refused a blood draw had moderate AP. The remaining 16 discharge samples were successfully processed for NMR testing.

Ultimately, 17 admission samples (10 mild AP, 5 moderate AP, and 2 severe AP) and 16 discharge samples (7 mild AP, 6 moderate AP, and 3 severe AP) were available for analysis.

### 3.3. Comparison of GlycA Levels between AP Patients and Healthy Controls

**Figure 1:**
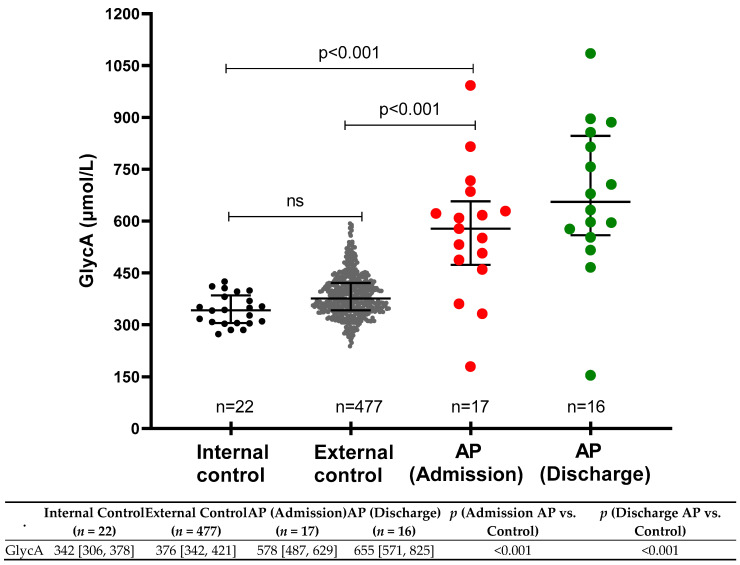
GlycA levels in healthy control subjects compared to patients with AP during hospitalization. ns = not significant.

GlycA levels in healthy controls and in AP patients on admission and discharge are shown in Figure 1. The median (IQR) GlycA level in healthy controls was 376 (342–431) μmol/L, and in AP patients, the median GlycA levels on admission and discharge were 578 (487–629) μmol/L and 655 (571–825) μmol/L, respectively. GlycA levels were significantly higher in AP patients than in healthy controls, both on admission and discharge (*p* < 0.001), but were not statistically different when admission and discharge GlycA levels were compared to each other (*p* = 0.121).

### 3.4. Subgroup Analyses of GlycA Levels Based on Severity

**Figure 2:**
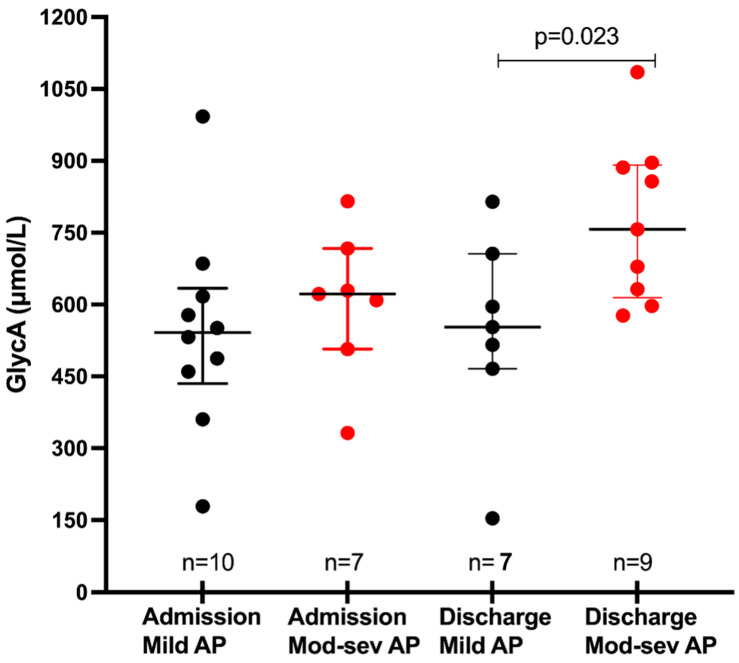
GlycA levels by severity of AP.

Given the smaller sample size, patients with moderate AP and severe AP were combined to form the moderate–severe AP group. We then compared admission and discharge GlycA levels between patients with mild AP and moderate–severe AP. Of the 10 patients in this moderate–severe AP group, admission GlycA levels were available for 7 patients (three samples were excluded due to disruption of specimen integrity). While GlycA levels at admission did not differ between the mild and moderate–severe AP groups, patients with moderate–severe AP showed significantly higher levels of GlycA on discharge compared to those with mild AP (533 ± 160 vs. 757 ± 254 μmol/L, *p* = 0.023) (Figure 2). Given that all 10 patients in the moderate–severe AP group had some degree of end-organ damage (temporary for <48 h in the moderate AP group and persistent for > 48 h in the severe AP group), a comparison of GlycA levels between patients with and without end-organ damage was not performed, as it would not show results any different from those outlined in Figure 2. Notably, the mean duration of hospitalization was 3.8 days for patients with mild AP, 7.2 days for moderate AP, and 11.5 days for severe AP.

### 3.5. Multivariable Logistic Regression

We performed multivariable logistic regression to test for associations between GlycA levels over 400 μmol/L, the cutoff for significant GlycA elevation, and the risk of AP and to study the association of GlycA levels over 400 μmol/L with the severity of AP, as shown in Table 2. After adjusting for BMI, our results showed that compared to controls, patients with GlycA levels > 400 μmol/L had 6.9 times the odds of having AP of any severity (OR = 6.88; 95% CI, 2.07–32.2; *p* = 0.004) and 6.1 times the odds of having mild AP (OR = 6.12; 95% CI, 1.48–42.0; *p* = 0.025).

## 4. Discussion

In this prospective pilot study, we provide the first evidence that GlycA levels are elevated in patients with AP and can be used as a potential biomarker of systemic inflammation in the context of AP. In our study, GlycA levels were significantly higher in hospitalized AP patients, both at admission and at discharge, when compared to healthy controls. There were no significant differences in GlycA levels between mild and moderate–severe AP patients at the time of initial hospital admission. However, patients with moderate-to-severe AP had significantly higher GlycA levels compared to patients with mild AP at the time of their hospital discharge. Lastly, GlycA levels greater than 400 μmol/L were associated with significantly higher odds of having AP. No differences in GlycA levels were observed in regard to the different etiologies of AP.

Several newer biomarkers of AP have been reported over the past decade in an effort to better predict disease activity and severity. Some of these include mitochondrial DNA, microRNA, and several other isolated biomarkers identified via proteomic and metabolomic pathways [23,24,25,26,27]. While GlycA has not been studied in AP previously, the recognition of GlycA as an inflammatory biomarker of AP in our study makes pathophysiological sense because each of the five acute phase glycoproteins that comprise GlycA (α1 acid glycoprotein, haptoglobin, α1 antitrypsin, α1 antichymotrypsin, and transferrin) has been individually studied over the years and shown to be associated with AP [28,29,30,31,32,33]. For example, in a study of 82 patients with AP, Mucha et al. showed that certain transferrin isoforms were uniquely associated with disease activity and severity in AP, more so in the case of alcohol-related AP [33]. It was also observed in a group of 30 AP patients that sialylation of transferrin is reflective of the intensity of inflammation and could be used as a clinical marker of early severity prediction in AP [34]. Similarly, in a prospective study of 34 patients, Karsidag et al. showed that α1-antichymotrypsin is elevated in the first 24 h of AP and has good diagnostic value [31]. One of the benefits of GlycA is that it is a composite signal that exhibits less biological variability than any of its individual components or other inflammatory biomarkers, such as CRP [16]. Although we had only two cases of AIP in our cohort, cross-sectional imaging did not reveal any other lesions, such as pancreatic cystic lesions or intraductal papillary mucinous neoplasia, that have been previously reported in the setting of AIP [35]. Hence, in our cohort, the levels of GlycA detected in AP patients were entirely due to the benign process of AP and were not confounded by a co-existing neoplastic process.

GlycA has been extensively studied as a marker of systemic inflammation in atherosclerosis and has been shown to have strong associations with future risk of cardiovascular disease-associated morbidity and mortality [18,19,36,37,38]. Among gastrointestinal conditions involving inflammation, GlycA has only been studied in IBD. In a prospective study, Dierckx et al. showed that GlycA levels were elevated in 58 patients with IBD compared to healthy controls [20]. The authors also measured pre- and post-treatment GlycA levels and showed that a treatment-induced decrease in GlycA levels was associated with clinical and endoscopic disease healing as well as concurrent improvement in other biochemical markers (CRP and fecal calprotectin levels). While our study similarly showed elevated GlycA levels compared to healthy controls, we did not see a treatment-induced decrease in GlycA levels at the end of our patients’ hospitalization. However, this difference could be explained by the fact that the interval between the two time points at which we measured GlycA (admission and discharge) was relatively shorter (mean LOS 5.7 days) compared to the IBD study (23.7 weeks in patients with Crohn’s disease and 13.7 weeks in those with ulcerative colitis). While it would be interesting to see a treatment-responsive decrease in GlycA levels in AP patients, the feasibility of this would be dependent on long-term outpatient follow-up after complete resolution of symptoms. Conversely, AP patients with more severe disease had significantly higher GlycA levels at discharge compared to those with mild disease. The reason for this is not entirely clear, partly due to not knowing the exact half-life of GlycA. However, it is a later-stage acute-phase protein, and as such, the levels rise around day 3–4, peak around day 7, and then slowly decrease with time as the inflammation subsides. GlycA is also a marker of tissue damage; for example, the GlycA level increases after acute myocardial infarction. Given that the inflammation in moderate-to-severe AP is generally higher than that in mild disease, we believe that the peak GlycA level may not have been reached at the time of discharge, despite clinical improvement in these patients. 

We recognize several limitations of our study, one of which was the small sample size. However, this was a pilot study that was primarily performed to assess the feasibility of testing serum GlycA levels in AP patients as well as to identify any potential clinical correlation with the disease course. Further studies incorporating more AP patients with variable disease etiology and severity are required to validate our results. In our study, patients with moderate-to-severe AP had higher GlycA levels compared to patients with mild AP at the time of their hospital discharge, but no significant differences were observed between the two at the time of hospital admission. Additionally, GlycA levels greater than 400 μmol/L were associated with higher odds of having mild AP but not moderate-to-severe AP. This suggests that while GlycA could be used as a reliable marker of diagnosis and overall severity in AP, its role as an “early” predictor of severity remains limited, potentially owing to the small sample size. We recognize that our study was not adequately powered to detect significant differences. We also note that we do not have 30-day post-discharge samples to assess a downward trend and/or normalization of GlycA levels compared to the time of discharge. As stated above, it is possible that the peak GlycA level may not have been achieved at the time of discharge, making it difficult to definitively establish GlycA as a marker of disease activity or severity and to observe a treatment-responsive decrease in its level.

The biggest strength of this study is that it is the first to assess GlycA as a diagnostic biomarker of inflammation in patients with AP. A major advantage is that GlycA testing is available clinically, i.e., it can be ordered for patient care and monitoring. Prospective enrolment of our study patients and the presence of multiple healthy control groups for comparison are additional strengths of our study.

While there is a body of literature showing the clinical utility of GlycA in chronic diseases, much less is known about the clinical utility of GlycA in acute illnesses. GlycA levels have been shown to be very high in children with Kawasaki disease [39]. In these patients, GlycA levels are much higher than those observed in patients with acute illnesses of bacterial or viral origin. Despite abundant ongoing research in this area, little is known about the etiology of Kawasaki disease and the severe inflammation that occurs in these children well after a pathogenic culprit has been detected. GlycA is also associated with disease severity in pediatric patients with COVID-19. Similar to those with Kawasaki disease, children with multisystem inflammatory syndrome of children (MIS-C) due to SARS-CoV-2 infection also have extremely high levels of GlycA [40]. GlycA levels were also remarkably high in patients with AP in our study, much higher than had been observed in other studies except in studies of adults with sepsis. Given that GlycA levels may reflect long-term tissue damage, it would be interesting to know when patients actually recover from severe AP. Therefore, this pilot study provides data that supports additional studies with long-term outpatient follow-up after complete resolution of symptoms to ascertain how long it takes for AP to subside, especially in our patients who presented with severe AP.

In conclusion, our pilot study highlights the use of GlycA as a novel diagnostic biomarker of inflammation in patients with AP. Given the high morbidity and mortality associated with moderate-to-severe AP, further studies with larger sample sizes are required to investigate the role of GlycA as a diagnostic biomarker of AP and time-sensitive trends in response to treatment.

## Figures and Tables

**Table 1:** Demographic and clinical characteristics of AP patients and healthy controls enrolled in this study. Data are presented as the mean ± SD or percentages (n) for quantitative and qualitative variables, respectively.

	External Healthy Control (*n* = 477)	Internal Healthy Control (*n* = 22)	AP (*n* = 20)	*p* Value (External vs. AP)	*p* Value (Internal vs. AP)
Demographic characteristics					
Mean age in years (SD)	40.0 (12.9)	37.6 (11.1)	41.8 (11.6)	0.520	0.244
Mean BMI in kg/m^2^ (SD)	24.7 (3.74)	25.3 (3.67)	29.9 (4.65)	<0.001	0.001
Male sex	187 (39.5%)	11 (42.3%%)	12 (60%)	0.111	
Race				1.000	0.211
Non-White	162 (34.2%)	13 (59.1%)	7 (35%)		
White	311 (65.8)	9 (40.9%)	13 (65%)		
Clinical characteristics					
Etiology of AP:					
Alcohol	-	-	10 (50%)		
Biliary			3 (15%)		
Hypertriglyceridemia	-	-	3 (15%)		
Other	-	-	4 (20%)		
Severity of AP:					
Mild	-	-	10 (50.0%)		
Moderate	-	-	7 (35.0%)		
Severe	-	-	3 (15%)		
History of recurrence:					
Primary	-	-	10 (50.0%)		
Recurrent	-	-	10 (50.0%)		
Current Smoker	-	-	11 (55.0%)		
Alcohol use disorder	-	-	10 (50.0%)		
Diabetes mellitus	-	-	7 (35.0%)		

**Table 2:** Association between GlycA levels >400 umol/L and severity of AP.

Comparison	OR [95% CI]	*p* Value
GlycA cutoff = 400 µmol/L		
AP vs. control	6.88 [2.07, 32.2]	0.004
Mild AP vs. control	6.16 [1.48, 42.0]	0.025
Moderate–Severe AP vs. control	10.0 [1.47, 229.2]	0.050
Moderate–Severe vs. Mild AP	1.75 [0.13, 44.8]	0.680

Logistic regression following generalized linear models is adjusted for BMI.

## Data Availability

The data presented in this study are available in this article.
